# Fiber Bragg Gratings in CYTOP Fibers Embedded in a 3D-Printed Flexible Support for Assessment of Human–Robot Interaction Forces

**DOI:** 10.3390/ma11112305

**Published:** 2018-11-16

**Authors:** Arnaldo Leal-Junior, Antreas Theodosiou, Camilo Díaz, Carlos Marques, Maria José Pontes, Kyriacos Kalli, Anselmo Frizera-Neto

**Affiliations:** 1 Graduate Program of Electrical Engineering, Federal University of Espírito Santo, Vitória 29075-910, Brazil; c.rodriguez.2016@ieee.org (C.D.); mjpontes@ele.ufes.br (M.J.P.); frizera@ieee.org (A.F.-N.); 2 Photonics and Optical Sensing Research Laboratory, Cyprus University of Technology, 3036 Limassol, Cyprus; theodosiou.antreas@gmail.com (A.T.); kkalli@cytanet.com.cy (K.K.); 3 Instituto de Telecomunicações and I3N & Physics Department, Campus Universitário de Santiago, University of Aveiro, 3810-193 Aveiro, Portugal; carlos.marques@ua.pt

**Keywords:** fiber Bragg gratings, polymer optical fiber, wearable devices, soft materials, additive layer manufacturing

## Abstract

We developed a flexible support with embedded polymer optical fiber (POF) sensors for the assessment of human–robot interaction forces. The supports were fabricated with a three-dimensional (3D) printer, where an acrylonitrile butadiene styrene (ABS) rigid structure was used in the region of the support in which the exoskeleton was attached, whereas a thermoplastic polyurethane (TPU) flexible structure was printed in the region where the users placed their legs. In addition, fiber Bragg gratings (FBGs), inscribed in low-loss, cyclic, transparent, optical polymer (CYTOP) using the direct-write, plane-by-plane femtosecond laser inscription method, were embedded in the TPU structure. In this case, a 2-FBG array was embedded in two supports for human–robot interaction force assessment at two points on the users’ legs. Both FBG sensors were characterized with respect to temperature and force; additionally, the creep response of the polymer, where temperature influences the force sensitivity, was analyzed. Following the characterization, a compensation method for the creep and temperature influence was derived, showing relative errors below 4.5%. Such errors were lower than the ones obtained with similar sensors in previously published works. The instrumented support was attached to an exoskeleton for knee rehabilitation exercises, where the human–robot interaction forces were measured in flexion and extension cycles.

## 1. Introduction

Advances in medicine and improvements to quality of life have led to an increase in the life expectancy of the general population [1]. An ageing world population has placed demands on the use of assistive technology and in particular towards novel robotic assistance and rehabilitation devices, since increasing neurological injuries and weakness of skeletal muscles inhibit the independent movement and full recovery of the elderly once such injuries are sustained [2]. In these cases, robotic therapy has several advantages over conventional therapy, which include higher repeatability for rehabilitation exercises and quantitative feedback of the patient recovery [3]. An additional advantage of robotic therapy is the customization of rehabilitation training with the transition between passive, active-assisted, and active-resisted by the robot controller [4]. In recent years, a novel approach for wearable robotic devices based on flexible materials has been proposed to increase the compliance between the human and the robot [5], where it is possible to develop a robot with greater user-synchronization, the so-called human-in-the-loop design [6].

The development of flexible structures has been driven by novel manufacturing processes, and three-dimensional (3D) printing may be regarded as one of the manufacturing processes that have enabled the development of complex and custom-made structures and geometries for soft robotics [7]. 3D printing is an additive layer manufacturing (ALM) process in which hot or melted polymers are injected layer-upon-layer to form the desired structure [7]. In general, 3D printing also offers low cost and the possibility of recycling many waste materials, which makes it an environmentally friendly solution and an important technology for the third industrial revolution realized through digital manufacturing [8]. 3D printing technology is well-aligned with the requirements of soft robotics devices and was employed in the development of several soft robotics devices, summarized in [7].

For accurate control of wearable devices for gait assistance and customization of rehabilitation treatments, the robotic devices rely heavily on the sensors’ feedback. Hence, inaccuracies in the sensor system can harm the operation of such devices, especially in impedance/admittance controllers in which the user’s intended movement is acquired and is used as feedback for the controller assisting the movement accordingly [9]. One approach to obtain the users’ intended movement is by using Electromyography (EMG) sensors. Although these sensors present the advantage of direct measurement of the muscle activity in the region where the electrodes are placed, they suffer from high noise and need direct contact with the user’s skin; the installation is cumbersome and time-consuming [10]. In addition, EMG sensors need complex signal processing techniques, and the measured electrical potential is not directly related to the applied force [10]. For these reasons, an alternative approach for the sensory feedback is to place instead the sensors in the wearable robot. In this case, the control is made based on the human–robot interaction force [11]. Conventionally, the sensors for human–robot interaction-assessment are piezoelectric sensors, capacitive force sensors, and strain gauges. However, these sensors are sensitive to electromagnetic fields, lack robustness, and require careful installation [10]. These issues escalate in soft robotics devices, where the flexibility requirements can inhibit the application of numerous electronic sensors.

As an emerging alternative for instrumentation in different applications, polymer optical fibers (POFs) sensors have been used to measure several parameters, which include angle [12], pressure [13], temperature [14], humidity [15], force [16], strain [17], and acceleration [18]. POFs share many of the advantages of their silica counterparts such as electromagnetic field immunity, multiplexing capabilities, and compactness [19]. Additionally, POFs also have higher strain limits, lower Young’s modulus, fracture toughness, and higher impact resistance when compared with silica optical fibers [19]. For these reasons, POF sensors can be regarded as an interesting alternative for the instrumentation of wearable devices for rehabilitation [20] and gait assistance [21]. In addition, such advantages of POF sensors allow for their embedding in flexible materials [22] and in particular are well suited for 3D printed structures [23].

Among different techniques for optical fiber sensing, fiber Bragg gratings (FBGs) have attracted significant attention due to their multiplexing capabilities, which make it possible to inscribe long arrays with an increasing number of sensors in the same fiber [24]. FBGs are periodic modulations of the fiber core’s refractive index, and different gratings can be inscribed in the same fiber just by modifying the period of this refractive index modulation, with each grating acting as a wavelength selective filter, reflecting light at a discrete wavelength that is directly proportional to the inscribed period. Changes to the period as a result of external perturbations are recovered as wavelength-encoded data. This offers a huge advantage as the data is an absolute quantity and may be recovered even if the signal is temporarily interrupted. FBGs can be fabricated using the methods discussed in [25], through ultraviolet (UV) laser irradiation with a phase mask [26], or by direct writing in the fiber core using a femtosecond (fs) laser [27]. A lot of progress have been achieved in POFs made of different materials [28]. Among those materials for POFs, the cyclic transparent optical polymer (CYTOPs) offer advantages over other materials, such as poly methyl methacrylate (PMMA) and cyclic olefin copolymers (COCs), due to their lower optical losses, particularly in the 1550 nm wavelength region [29]. This advantage enables the employment of commercially available optical components, which are generally designed to work within the 1550 nm wavelength region. Thus, FBGs inscribed in CYTOP fibers are good candidates for practical sensing applications. However, such fibers present multimode operation, which can inhibit their application as sensors and in long arrays. Theodosiou et al. [30] proposed a direct-write, plane-by-plane inscription method with optimized inscription parameters using a fs laser, where it is possible to control the coupling between the grating and core modes, leading to a single peak spectrum, which can be regarded as an important advance towards practical sensing applications with FBGs inscribed in POF.

Considering (i) the necessity of novel sensing solutions for flexible structures, (ii) the advantages of 3D printing on the development of flexible structures and sensors’ embedment, and (iii) the advances in the inscription of long FBG arrays in CYTOP fibers, this paper presents the development of a flexible support for human–robot interaction force assessment using a FBG array in CYTOP fibers. Two exoskeleton supports were 3D printed with materials that have different degrees of flexibility, namely acrylonitrile butadiene styrene (ABS) and thermoplastic polyurethane (TPU). The structure that was attached to the exoskeleton was made of ABS for higher resistance, whereas each FBG of the 2-FBGs array was embedded in structures made of TPU for higher flexibility. Then, each FBG was characterized with respect to temperature and force. Following the sensor validation, the FBG-embedded, 3D-printed flexible supports were applied on a lower limb exoskeleton for knee rehabilitation exercises. In this way, the contribution of this paper is in the development and characterization of FBG-embedded flexible structures for the next generation of wearable robots for rehabilitation.

Following the introduction presented in this section, the remainder of the paper is organized as follows. Section 2 depicts the design of the proposed 3D-printed, FBG-embedded shank supports. Then, Section 3 shows the experimental setup used in the sensor characterization and validations as well as the lower limb exoskeleton, where the proposed shank support was applied. Results and discussions of the sensors responses in characterization and validation tests are depicted in Section 4. Finally, concluding remarks and future investigations are presented in Section 5.

## 2. 3D-Printed, FBG-Embedded Flexible Supports Design

For the 3D printed, FBG-embedded flexible support design, the first step was the grating inscription in a commercial gradient index multimode CYTOP fiber (Chromis Fiberoptics Inc, Warren, NJ, USA). More specifically, the fiber had a core diameter of 120 µm, a cladding thickness of 20 µm, and a polycarbonate overcladding. Regarding the equipment for grating inscription, an air-bearing translation stage with nanometer precision was used to position the fiber for direct write, plane-by-plane inscription using an fs laser. The inscription was performed with an fs laser operating at 517 nm (repetition rate of 5 kHz and pulse energy of ~80 nJ) with 220 fs pulse duration (HighQ laser femtoREGEN), and the laser beam was focused with a ×50 objective lens. In this way, the refractive index modifications were induced at the center of the fiber core. The second step for the flexible support design was the fiber annealing in which the POF was positioned inside a climate chamber 1/400 ND (Ethik Technology, Vargem Grande Paulista, Brazil) at a temperature of 70 °C for approximately 12 h. Such heat treatment promotes the relaxation of the polymer’s molecular alignment, which leads to the reduction of the internal stress created in the fiber manufacturing process [31]. Thus, it results in the reduction of the fiber Young’s modulus, which leads to the increase of the sensor force sensitivity [32]. In addition, the annealing in CYTOP fibers also results in the reduction of the sensor hysteresis [32].

**Figure 1:**
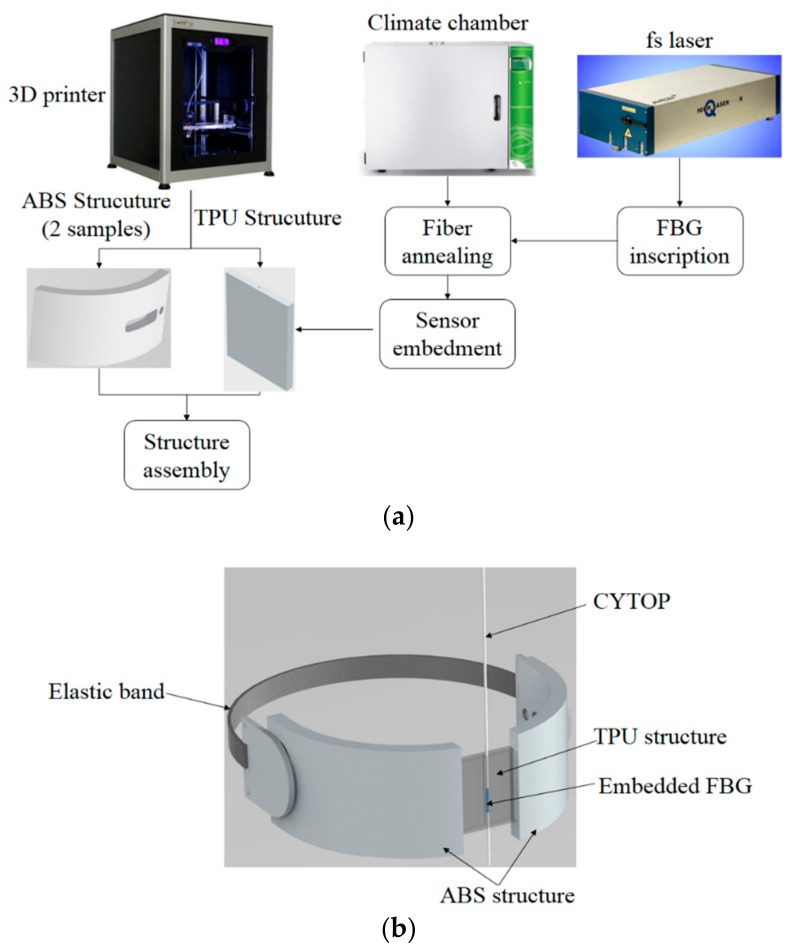
(**a**) Block diagram of the proposed method for the three-dimensional (3D)-printed, fiber Bragg grating (FBG)-embedded flexible support fabrication; (**b**) Schematic representation of the assembled flexible support. ABS = acrylonitrile butadiene styrene; TPU = thermoplastic polyurethane; fs = femtosecond; CYTOP = cyclic, transparent, optical polymer.

The 3D-printed structures were fabricated using the 3D printer Sethi3D S3 (Sethi, Campinas, Brazil), where the structures had a predefined infill density, which indicates the amount of employed material to fill the 3D-printed structure. For the ABS section of the support (Figure 1), the infill density was 70% in order to increase the resistance of the structure, since it is attached to the exoskeleton. Two ABS structures were printed for each support, where the elastic bands for the attachment of the support on the user’s leg are positioned. In between the ABS structures, a flexible structure made of TPU was placed, and the FBGs were embedded in each TPU structure. The proposed method for the exoskeleton support fabrication provides stability in the interface between the robotic device and the user. At the same time, the flexibility of the support provides a comfortable and compliant support for the user’s leg. Figure 1a shows the schematic representation and block diagram for the 3D-printed flexible support fabrication, where it is possible to customize the support dimensions for each user. In addition, Figure 1b shows the schematic representation of the assembled support.

**Figure 2:**
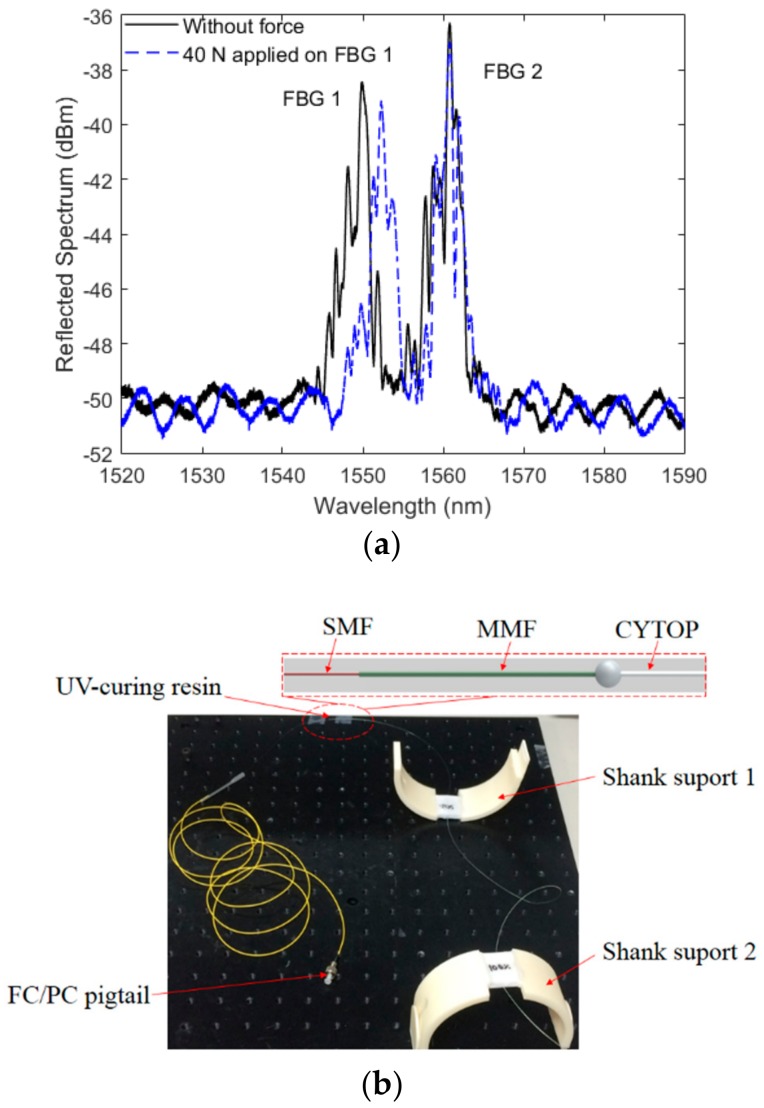
(**a**) Reflected spectrum of the FBG array without applied force and after a transverse force application of 40 N and (**b**) Picture of the fabricated flexible shank supports with embedded FBGs (without the elastic bands). Figure inset shows the schematic representation of the fiber-connectoriztion method. SMF = single mode fiber; MMF = multimode silica fiber, FC = ferrule connector; PC = physical contact.

The method shown in Figure 1 was used to fabricate each support, the support was assembled with the aid of a thermoplastic resin. In order to evaluate the repeatability of this approach, the multiplexing capabilities of the FBGs, and to provide higher stability for the user’s leg, an array with two FBGs was inscribed in CYTOP using the aforementioned materials and methods. The FBGs spectra after the annealing are shown in Figure 2a, where the spectrum was acquired using a Micron Optics sm125 FBG interrogator. However, this interrogator has low acquisition frequency (2 Hz), which is unsuitable for the proposed application. For this reason, for the tests with the proposed sensors, the spectrometer I-MON 512 (Ibsen Photonics, Farum, Denmark) was used. In this case, the peak detection was made through a Gaussian fit on the spectra and the peaks were identified after setting a threshold. In addition, it was also possible to change the integration time of the device, which could act as a low pass filter for the spectra, reducing the side lobes of the spectra that makes the single peak detection easier. Although there were a few distortions on the spectrum when the transverse force was applied due to birefringence and polarization effects, the spectrum moved in unison as shown in Figure 2a for a transverse force of 40 N on FBG 1. Thus, by applying the aforementioned methodology, a single peak was detected for each FBG at about 1550 nm for FBG 1 and 1560 nm for FBG 2. The picture of the exoskeleton’s support (that was positioned on the user’s shank region) following the FBG array-embedment is shown in Figure 2b.

The POF connectorizationwas made using a UV-curing resin that, after the proper alignment, connected the POF to a multimode silica fiber (MMF). The MMF was subsequently fusion-spliced to a single mode fiber (SMF) using a commercial fusion splicer machine (Fujikura, Tokyo, Japan). In this way, it was possible to guarantee a smooth diameter reduction between POF and SMF and also provide a stable connection between the FBG inscribed in CYTOP and the interrogator.

## 3. Experimental Setup

For a FBG sensor, the shift in the Bragg wavelength (Δ*λ_B_*) is correlated with the measured parameter. As is well-known, FBGs are inherently sensitive to temperature and strain variations following Equation (1).

(1) $$\Delta \lambda_{B}=\left[\left(1-P_{e}\right)\varepsilon +\left(\alpha +\xi \right)\Delta T\right]\lambda_{B}$$

where *P_e_* is the photo-elastic constant, *ε* is the strain, Δ*T* is the temperature variation, *λ_B_* is the Bragg wavelength, *α* is the material thermal expansion coefficient, and *ξ* is the thermo-optic coefficient. However, other physical parameters that influence the strain or temperature variations in the fiber also result in the wavelength shift. One of these parameters is the force, which leads to a stress (*σ*) on the fiber that is related to the strain through the well-known Hooke’s law described in the elastic limit by Equation (2) as follows:

(2) σ=Eε

Regarding Equation (2), the term *E* is the fiber Young’s modulus. For polymers, the Young’s modulus changes as a function of the temperature. Thus, considering Equations (1) and (2), the temperature variation leads to a wavelength shift due to the second term of the sum inside the bracket. In addition, it also leads to a variation of the sensor stress/force sensitivity [33].

This theoretical background has led to the development of the characterization methodology for the FBG-embedded force sensors, for which the sensors are characterized with respect to force, temperature, and the simultaneous variation of both parameters. One may now evaluate the force sensitivity variation as a function of temperature, and enable the compensation of the temperature’s influence on the sensor force response. In addition, polymers are viscoelastic materials, which naturally do not have constant response to stress and strain [34]. The polymer response under a constant stress or strain is characterized by a creep (or recovery) that is described as a time-dependent viscous deformation when loading is applied (or removed) [34]. Such an effect was characterized for a PMMA fiber [35], for a long period grating [36], and an FBG [37]. In addition, a compensation method for such an effect was proposed in [38], where an exponential regression was applied on the creep response, which was compensated for by dividing the sensor response to the characterized time constant. It is worth mentioning that the transverse force applied on the FBG also leads to birefringence and polarization effects, which cause a polarization dependent loss that increases linearly with the transverse force. Even though this principle was used on the development of transverse force sensors [16], we considered only the wavelength shift caused by the applied force as shown in previous reported works for pressure [22] and force [33] sensors based on FBGs.

**Figure 3:**
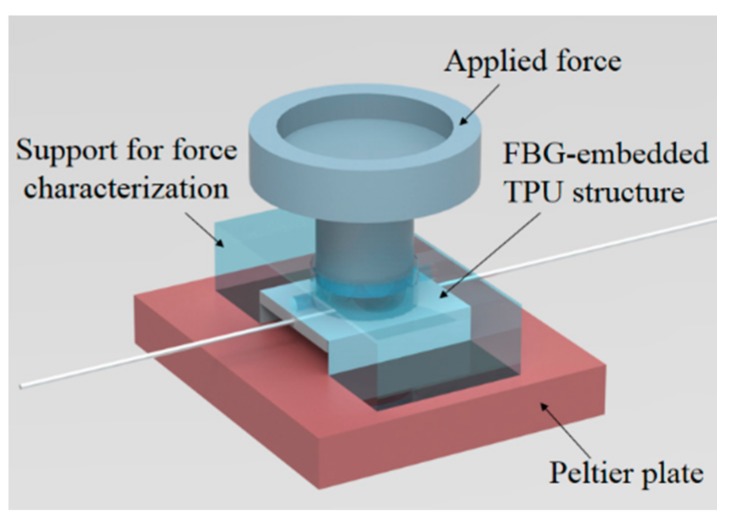
Experimental setup for the FBG sensors (embedded in the TPU structure) characterization.

As the POF was embedded in another polymer (TPU structure) the tests for the sensors’ characterization were made for the whole structure in which the sensor was embedded. For this reason, each TPU structure (Figure 1) with embedded FBGs was placed on the experimental setup shown in Figure 3. The force was applied using calibrated weights positioned in the region indicated in Figure 3, where the whole force was transmitted to the embedded FBG. These weights had a known mass and, thus, the applied force was also known. The range for the force characterization was up to 40 N (in 10 N steps) with a stabilization time of 5 min, whereas for the creep response characterization, a force of 20 N was applied, and the sensor response was monitored over 10 min, and the time constant of the FBG-embedded structure was experimentally obtained. For the temperature characterization, the TPU structure (with the setup for force characterization) was positioned on top of a Peltier plate (TEC1-12706, Hebei I.T. Co. Ltd., Shanghai, China), which had its temperature controlled by a temperature controller (TED 200C, Thorlabs, Newton, NJ, USA). The range of the temperature test was from 25 °C to 45 °C, in steps of 5 °C with a stabilization time of about 5 min. The temperature range was chosen to ensure human comfort as described in [10], given that the proposed support was in contact with the user. Additional force characterization tests were made at different temperatures (30 °C, 35 °C, 40 °C) to evaluate the sensitivity variation of the force sensor. Hence, by considering the sensitivity variation as a function of the temperature, it was possible to compensate for the temperature effects on the sensor response, as described in [33].

**Figure 4:**
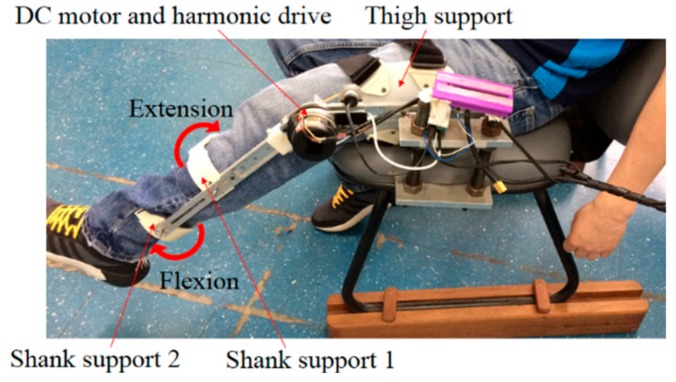
Exoskeleton for knee rehabilitation exercises with the 3D-printed flexible supports positioned on the shank region.

Following the characterization and validation of each FBG, the 3D-printed, FBG-embedded flexible support (shown in Figure 2b) was positioned as the shank supports of a lower limb exoskeleton for knee rehabilitation, as show in Figure 4. The supports were positioned such that when the flexion cycle was made, the highest force was applied to the shank support 1, whereas in the extension cycle, shank support 2 was subjected to the highest force. In addition, the configuration of the shank supports shown in Figure 4 provided higher stability for the user’s leg during the rehabilitation exercise. Moreover, the support flexibility provided comfort for the user. Regarding the exoskeleton structure, it was placed on a chair as shown in Figure 4, where a DC (Direct current) motor with a harmonic drive was responsible for the control of the flexion/extension movements. A thigh support was used to position and align the user’s thigh and the robot structure, whereas the proposed 3D-printed flexible supports were placed on the user’s shank. For the tests with the exoskeleton, sequential flexion/extension cycles were performed, and the response of each FBG was acquired by the FBG spectrometer I-MON 512 (Ibsen Photonics, Farum, Denmark) for a 10-kHz acquisition frequency. Furthermore, the exoskeleton provided three sets of rehabilitation exercises: active-assisted, passive, and active-resisted movements. In the active-assisted movement, the exoskeleton motor helped the user on the flexion/extension movement task, whereas the opposite occurred in the active-resisted mode, i.e., the impedance of the robot increased the difficulty in the movement. The passive operation mode (and the one used in the validation tests) may be regarded as an intermediate operation mode, where the motor did not provide any assistance or difficulty for movement. Thus, the impedance in the flexion/extension cycles were due to friction and back-drivability of the wearable robot.

## 4. Results and Discussion

**Figure 5:**
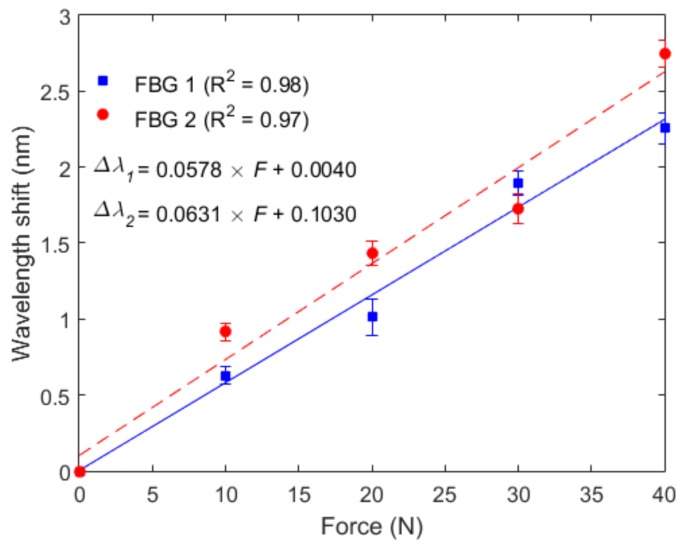
Force characterization of FBG 1 (embedded in the shank flexible support 1) and FBG 2 (embedded in the shank flexible support 2) at constant temperature of 25 °C.

The results obtained in the force characterization at a constant temperature of 25 °C for FBG 1 and FBG 2 (embedded in shank support 1 and 2, respectively) are presented in Figure 5, where the sensitivities of 57.8 pm/N and 63.1 pm/N were obtained for FBG 1 and FBG 2, respectively. In addition, the equations presented in Figure 5 were obtained through the linear regression between the applied force and wavelength shift for FBGs 1 and 2. Compared to previous work for which the force was directly applied on the fiber (without the 3D-printed structure), the obtained sensitivity was lower than the one without the TPU structure [32]. The reason for this behavior is the reduction of the strain transmitted to the fiber when it is embedded in a different structure. Moreover, the structure also influenced the sensor linearity, which may have been lower as a result of nonlinearities in the strain distribution along the TPU structure that was transmitted to the fiber. Comparing FBGs 1 and 2, the higher sensitivity of FBG 2 can be related either to the embedment process in the TPU structure or the differences on the material properties along the fiber, which may have resulted from anisotropy and the annealing conditions. Although, the FBG 1 had lower force sensitivity, it also had higher linearity, which leads to lower errors in the force estimation.

**Figure 6:**
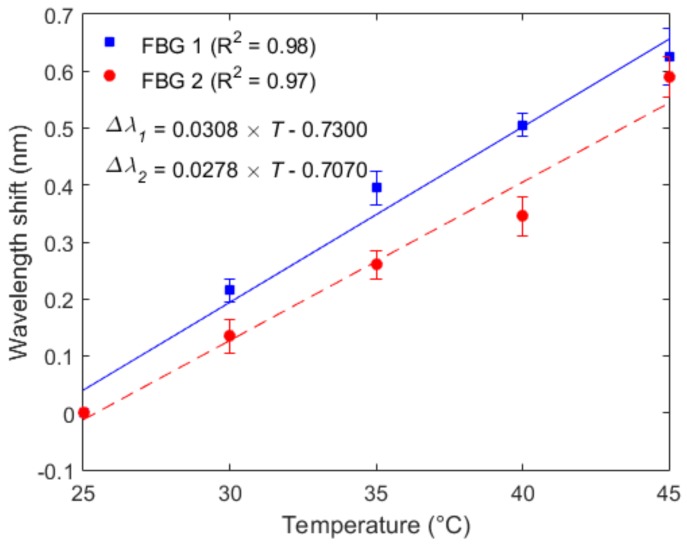
Temperature characterization of FBG 1 (embedded in the shank flexible support 1) and FBG 2 (embedded in the shank flexible support 2).

The second step in the sensors’ characterization was their temperature analysis without the influence of force. In this case, it was possible to estimate the offset that the temperature caused in the sensor’s response. Figure 6 shows the results of such temperature characterization for both FBGs. In contrast with the results obtained in the force characterization, the temperature sensitivities for FBGs 1 and 2 (30.8 pm/°C, 27.8 pm/°C, respectively) were higher compared with fibers that were not been embedded [32,39]. Since the temperature increase also lead to a thermal expansion of the TPU structure, the thermally-induced strain in the structure was directly transmitted to the FBG, which lead to an additional wavelength shift due to the TPU structure’s thermal expansion, resulting in higher temperature sensitivity for the embedded sensor.

**Figure 7:**
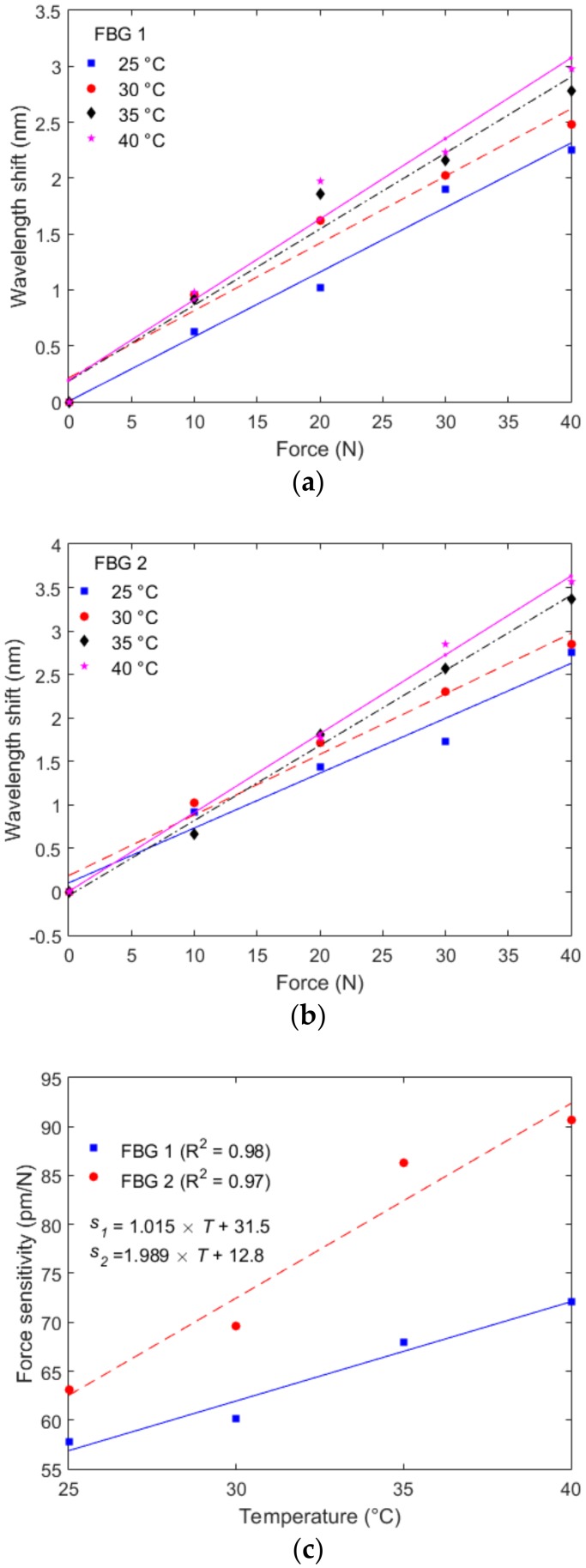
(**a**) Force characterization at different temperatures for FBG 1; (**b**) Force characterization at different temperatures for FBG 2 and (**c**) Force sensitivity as a function of the temperature for FBGs 1 and 2.

After the temperature and force characterizations, each sensor was subjected to applied forces at different temperatures in order to evaluate the force sensitivity variation as a function of temperature. Thus, the FBGs were characterized for different temperatures (30 °C, 35 °C, and 40 °C) with the same force range (0 to 40 N) as used for the test at room temperature (25 °C). The results of the characterization at different temperatures for FBG 1 and 2 are presented in Figure 7a,b, respectively. In addition, the force sensitivity of both FBGs at each temperature is shown in Figure 7c. The results show an increase in the sensor sensitivity when the force is applied at higher temperature conditions, which is expected since the Young’s modulus of the fiber is reduced as the temperature increases [33]. Thus, by combining Equations (1) and (2), we observe that the temperature increase reduced the Young’s modulus, which caused an increase in the sensor sensitivity to stress (or force). However, the POF was embedded in a structure made of another polymer (TPU, in this case), which also has its own Young’s modulus dependency with temperature. For this reason, the sensitivity variation with respect to the temperature was different when compared with unembedded CYTOP (shown in [33]). In this case, a linear regression for the sensors’ force sensitivities was obtained for each FBG, where the coefficients presented in Figure 7c show a higher temperature dependency on the force response of FBG 2, which can be related to minor differences in the embedment conditions.

**Figure 8:**
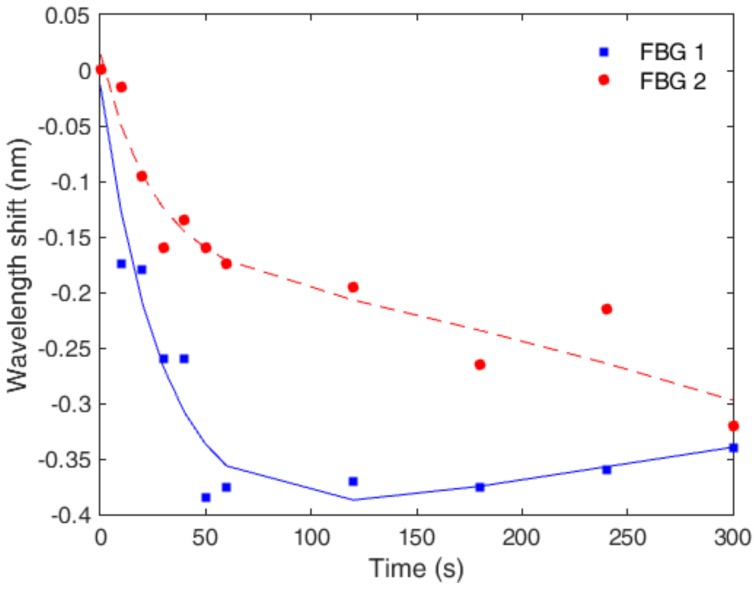
Creep response and exponential regression for FBGs 1 and 2 for 5 min.

For the last set of characterization tests, the FBGs were positioned on the experimental setup of Figure 3 and a constant force was applied on each FBG at constant temperature of ~22 °C (room temperature). The sensor responsewas monitored for 5 min, and an exponential regression was performed on the response of each FBG, as shown in Figure 8. The creep response characterization of FBGs 1 and 2 was not made at the same time due to operational limitations. Thus, the reason for the slight increase on the wavelength shift of FBG 1 is related to an increase of room temperature of about 0.9 °C on its characterization. In addition, if the material is thermorheologically simple, temperature variations will only lead to an offset on the polymer viscoelastic response [34]. It was demonstrated in [35] that PMMA behaves as a thermorheologically simple material in the time and temperature ranges used on the sensor characterization. Since PMMA and CYTOP have similarities in molecular structure, one can assume for simplification that the CYTOP is also a thermorheologically simple material, and the temperature variations will result in an offset of the material response.

**Figure 9:**
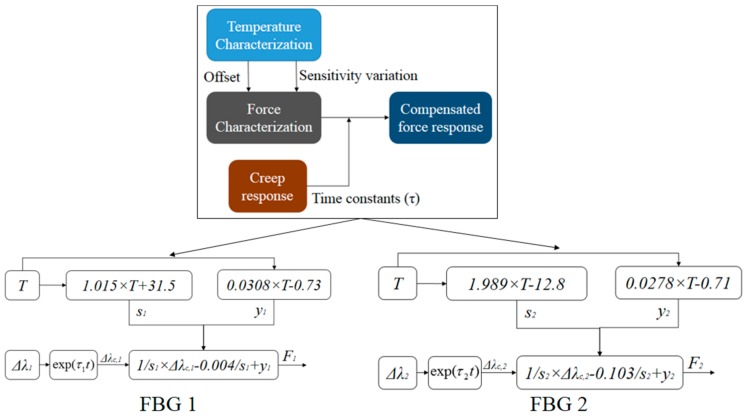
Block diagram of the force estimation for FBGs 1 and 2 with compensation of creep and temperature effects.

Applying the results and regressions of each characterization, one can obtain a characterization equation for each FBG in which the effects of creep and temperature are compensated. For this reason, the linear regressions for force response at room temperature for FBGs 1 and 2 were applied with the isolation of the force term (see Figure 5). Then, the compensation for the creep response was applied in the measured wavelength shift (Δ*λ_C_*), whereas the force sensitivity as a function of the temperature, shown in Figure 7c, were also applied. The offset in the force response due to the temperature (*y*) was also considered by means of applying the temperature regression presented in Figure 6. A block diagram with the approach for obtaining the calibration equation of each sensor as well as the equation for the estimation of the compensated force (for each FBG) is depicted in Figure 9, where *τ_1_* and *τ_2_* are the time constants for FBG 1 and 2, respectively.

**Figure 10:**
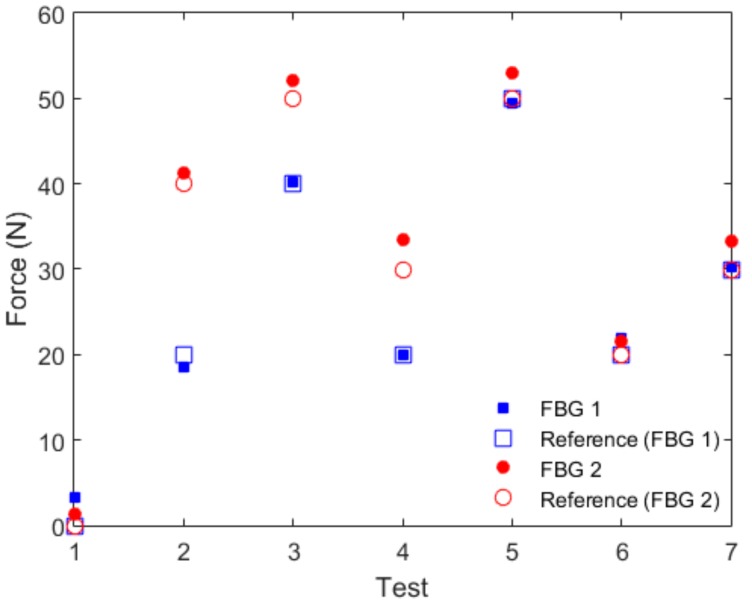
Validation tests for FBGs 1 and 2 at different forces and temperatures, where tests 1–3 were made at 25 °C, 4 and 5 at 35 °C, and the remainder (6 and 7) at 45 °C.

In order to validate the sensor, additional tests were made under different temperatures and applied forces on both FBGs 1 and 2, as shown in Figure 10. In this validation, seven tests were made with different forces and temperatures, where tests 1 to 3 were made at room temperature (25 °C). The temperature was increased to 35 °C and tests 4 and 5 were performed, whereas the tests 6 and 7 were made at 45 °C. The forces applied for each test are shown in Figure 10 as the ‘reference’ for each test and are compared with the responses of FBGs 1 and 2. The comparison showed a root mean squared error (RMSE) of ~0.50 N for FBG 1 and ~2.29 N for FBG 2. The reason for the higher RMSE for FBG 2 was the lower sensor linearity, as presented in the characterization tests, which lead to higher errors in the force estimation when the linear regression was made. Nevertheless, the relative error for FBG 2 (considering the whole force range of the test) was 4.5%, which is within the accuracy range of some conventional technologies for exoskeleton instrumentation [10], but with the additional advantages of electromagnetic fields immunity, compactness, flexibility, and multiplexing capabilities. In the case of FBG 1, the errors were even lower, since a relative error of 1.0% was obtained, where such a low error shows the possibility of achieving POF sensors with greater accuracy than the conventional technologies for robotic instrumentation. It is worth mentioning that such low errors were obtained by the combination of the different characterization tests performed, which resulted in an equation that compensated for different aspects of the sensor response, i.e., temperature cross-sensitivities and creep responses were obtained. Thus, it was expected that the results presented in Figure 10 with the compensation equations would present lower errors than the ones obtained in the characterization tests, where none of the compensation equations were applied.

**Figure 11:**
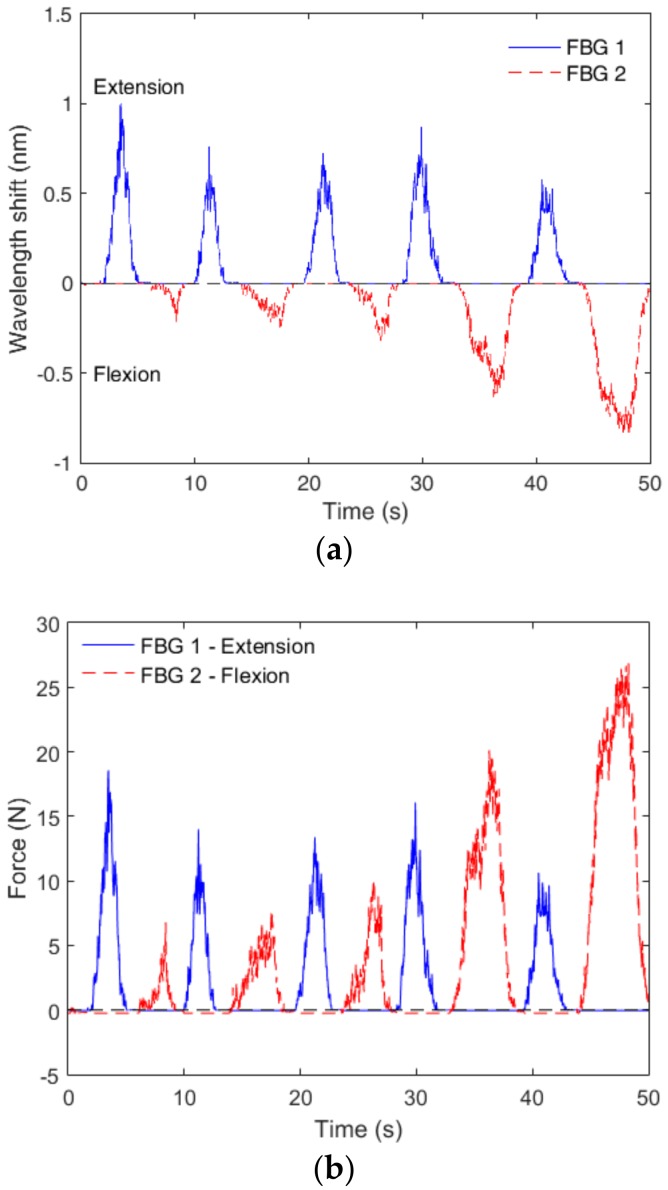
(**a**) Responses of the FBGs embedded in shank support 1 and 2 for extension (FBG 1) and flexion (FBG 2) cycles and (**b**) Human–robot interaction force measurement with the FBG-embedded, 3D-printed flexible supports.

Following the sensors’ characterization and validation, they were positioned on the exoskeleton (Figure 4) for the application as sensors for human–robot interaction forces, where the user was positioned on the chair with the thigh and shank supports attached. The user was asked to perform flexion and extension cycles. The results for five flexion/extension cycles are presented in Figure 11, where Figure 11a shows the response of FBGs 1 and 2 and Figure 11b presents the estimated force using the equations presented on the block diagram of Figure 9. The response of FBG 2 is inverted to help clarify the phase difference between both sensors, where the FBG 1 (shank support 1) is activated at extension movements and FBG 2 (shank support 2) shows a wavelength shift when the flexion cycle occurs. Regarding the force estimation presented in Figure 11b, the extension cycles presented similar behavior with the measured forces between 10 N and 15 N, which are similar to results previously obtained with electronic and optical fiber sensors [40]. Additionally, the human–robot interaction forces in flexion cycles had higher variation, where the user applied more force in the exoskeleton as the test progressed. This behavior was related to variations of the user interaction with the exoskeleton that generally varied between different users. Nevertheless, such increases in the interaction forces as the test progressed were also verified in previous work [40]. Thus, the tests with the FBG-embedded shank supports attached to the exoskeleton showed the feasibility of the proposed approach on the human–robot interaction forces assessment. For this reason, the 3D-printed FBG-embedded supports can be regarded as a viable option not only for the monitoring and validation of control techniques for the minimization of human–robot interaction forces [41] but also in the instrumentation of soft robotics wearable devices [5].

## 5. Conclusions

This paper presented the design and sensors’ characterization of a 3D-printed, FBG-embedded flexible support for human–robot interaction force assessment in a wearable exoskeleton. The principal aims were user comfort, ease of installation, and flexibility, therefore, part of the support was designed with a flexible structure made of TPU, where the FBGs were embedded. The part of the support that was attached to the exoskeleton was made of ABS to provide higher stability in the interface between the user and the robotic device. Following the support fabrication, the FBGs embedded in the TPU structure were characterized with respect to force, temperature, and the combination of both parameters. In addition, the creep response (which naturally occurs with viscoelastic materials such as the CYTOP) was also characterized. A method for the compensation of the effects of temperature and creep in the force response was proposed, and the validation tests showed errors between 2.29 N (4.5%) in the worst case and 0.50 N (1%) in the best case. The proposed FBG-embedded 3D-printed supports were attached on the shank region of an exoskeleton for knee rehabilitation for the human–robot force assessment, where the results showed the feasibility of the proposed approach. The device used on the sensors applications was employed for knee rehabilitation, which means that this device was fixed on a chair and it was not used for gait assistance. Even though this feature reduces the constraints of portability for the FBG interrogator, it is important to mention that many works have proposed low cost and portable interrogators [15,42], where even FBG interrogators on chips have been studied [43]. In addition, there are also commercially available FBG transceivers with higher portability [44]. Therefore, with these advantages of FBG interrogation technologies, it is possible to foresee the application of FBG sensors on the instrumentation of wearable devices for gait assistance outside the clinical environment. Following the positive feedback of the proposed approach, future research will involve the application of the proposed instrumented support in fully-flexible robots. In addition, future investigations will include using the FBG sensor technology for the complete instrumentation of the robot by developing sensors for temperature, pressure, humidity, and angle, taking into account all the requirements for a wearable robot’s instrumentation.
